# Application of X‐Ray Absorption Spectroscopy in Electrocatalytic Water Splitting and CO_2_ Reduction

**DOI:** 10.1002/smsc.202100023

**Published:** 2021-08-03

**Authors:** Bin Wang, Shengqi Chu, Lirong Zheng, Xiaodong Li, Jiangwei Zhang, Fuxiang Zhang

**Affiliations:** ^1^ State Key Laboratory of Catalysis Dalian Institute of Chemical Physics Chinese Academy of Sciences; Dalian National Laboratory for Clean Energy The Collaborative Innovation Center of Chemistry for Energy Materials (iChEM) Zhongshan Road 457 Dalian 116023 China; ^2^ Center for Advanced Materials Research Zhongyuan University of Technology Zhengzhou 450007 China; ^3^ Beijing Synchrotron Radiation Facility Institute of High Energy Physics Chinese Academy of Sciences Beijing 100049 China

**Keywords:** CO_2_ reduction mechanisms, water splitting, X-ray absorption spectroscopy

## Abstract

Renewable energy conversion and storage are crucial in aiding the worldwide efforts to replace fossil fuels and achieve carbon neutrality. In particular, electrocatalytic water splitting to obtain H_2_ and CO_2_ reduction to high‐value‐added fuels can lead to an epoch of hydrogen energy and a closed‐loop carbon cycle. Although many reported electrocatalysts have demonstrated outstanding performance at the laboratory scale, the origin of the superior electrocatalytic activity and selectivity and the structural evolution of these catalysts remain ambiguous. For this purpose, operando techniques are able to combine the characterizations of the catalyst with measurement of its performance under real working conditions. Herein, a critical overview of recent developments in understanding the underlying mechanisms of water splitting and CO_2_ reduction is presented, emphasizing the advanced operando X‐ray absorption spectroscopy characterization of these two electrocatalytic reactions. Some perceptions of the rising X‐ray technique directions and the foreground in this field are also presented.

## Graphical abstract

A brief introduction on the application of X‐ray absorption spectroscopy (ex situ and in situ) in electrocatalytic water splitting and carbon dioxide reduction is systematically given, aiming to understand the structural evolution of electrocatalysts and plausible mechanisms in modulating the electronic and geometrical structures to improve the electrocatalytic activity and selectivity of these two reactions.

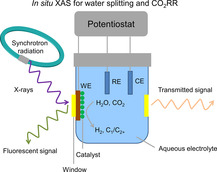

## 1 Introduction

Small molecules such as H_2_O, H_2_, O_2_, and CO_2_ play a key role in climate change, the energy crisis, and environmental issues.^[^ ^1^, ^2^, ^3^, ^4^, ^5^ ^]^ Many of these small molecules are typically generated or transformed in force in the chemical industry. Because they are involved in many elementary reactions relevant to efficient energy storage and conversion via the recombination of chemical bonds, they are essential parts of all technological roadmaps for edging out current fossil energy structures. Intense efforts are being dedicated to transforming these small molecules into high‐value‐added chemical fuels.^[^ ^2^, ^6^ ^]^ However, mastering the chemical nature of these small molecules remains a significant challenge.

These small molecules are relatively chemically stable; however, their chemical transformations are thermodynamically energy‐consuming. In addition, such transformations involve a multistep electron process, typically coupled with proton transfer, in which the reaction pathways are diverse and complex. Unraveling the underlying mechanism of such reactions is a daunting challenge to chemical researchers, and addressing the challenge is crucial for the rational design and construction of catalysts.^[^ ^7^, ^8^, ^9^, ^10^ ^]^ Inspired by nature, researchers use metals to activate these relatively stable molecules and modulate their reactivity in various ways, including thermocatalysis,^[^ ^11^ ^]^ photocatalysis,^[^ ^12^ ^]^ and electrocatalysis.^[^ ^8^ ^]^

**Figure 1:**
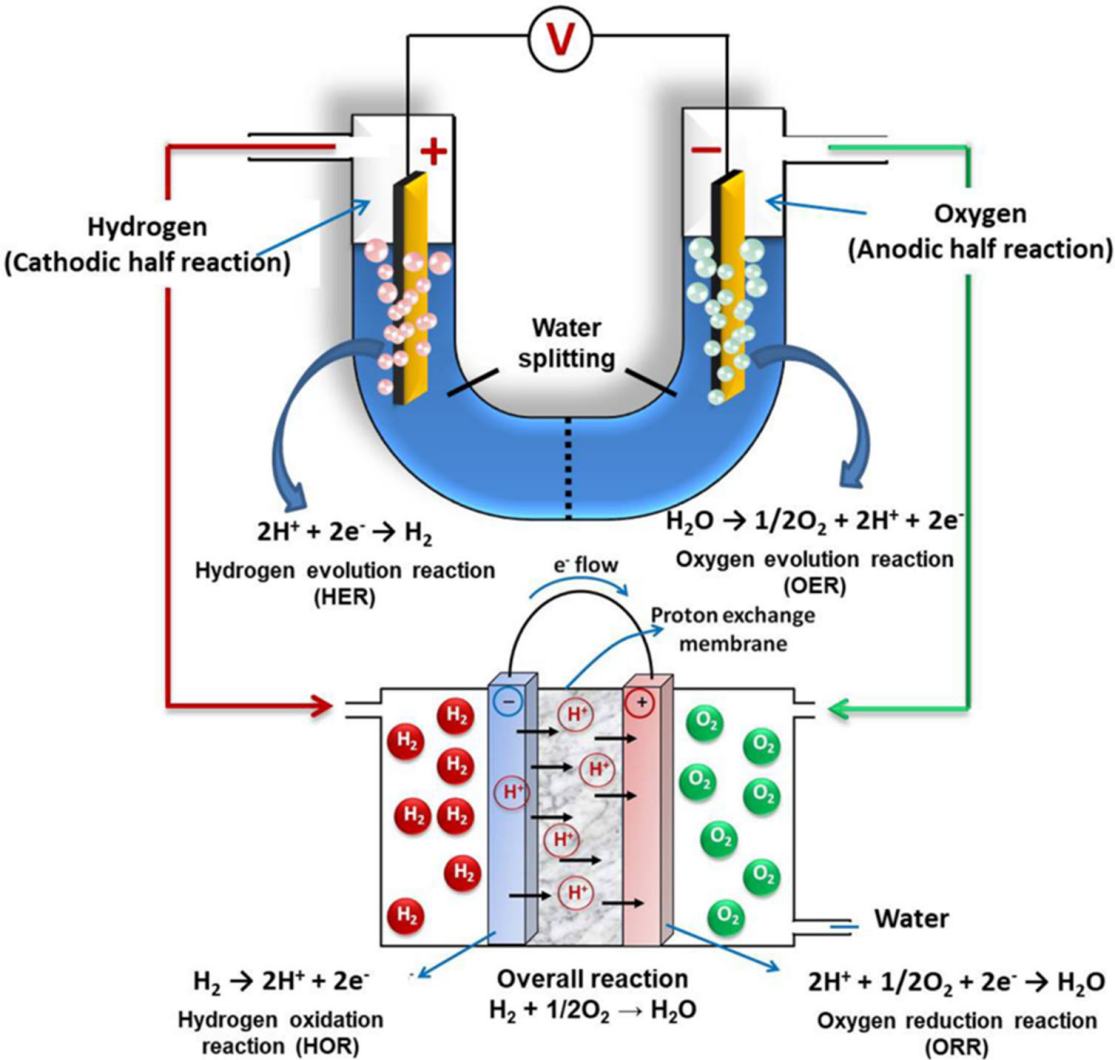
Schematic representation of small molecules (H_2_O, H_2_, and O_2_) involved in energy conversion applications: hydrogen evolution and oxygen production via water hydrolysis and hydrogen oxidation and oxygen reduction in the fuel cell. Reproduced with permission.^[^ ^17^ ^]^ Copyright 2017, American Chemical Society.

Electrocatalysis is the core of many clean energy conversion and storage systems. Developing highly efficient electrocatalysts to boost the activation and reactivity of these small molecules is an important research interest.^[^ ^13^, ^14^ ^]^ To date, enormous electrocatalysts have been reported that exhibited excellent performance in electrocatalytic water splitting as well as the CO_2_ reduction reaction (CO_2_RR). Electrocatalytic water splitting can be divided into two half‐reactions—namely, the hydrogen evolution reaction (HER) and oxygen production reaction oxygen evolution reaction (OER)—and it is a promising technical route to replace fossil fuels (**Figure** **1**). In acidic water splitting, the HER occurs with fast electrode reaction kinetics. In contrast, the OER is severely sluggish in kinetics because of its involved four‐electron process, resulting in a high overpotential for the entire water splitting. The high reaction kinetic barriers of the OER significantly impede the overall efficiency of the process, which limits the large‐scale industrial application of water splitting.^[^ ^15^, ^16^ ^]^ Moreover, only limited OER electrocatalysts, such as Ir/Ru‐based precious metal materials, have been exploited in acidic media. There is an additional issue in alkaline electrolysis of water, for example, the activation of water molecules and hydroxyl desorption required for hydrogen evolution.^[^ ^17^ ^]^ Furthermore, the OER performance of catalysts significantly affects the efficiency of many metal–air batteries and fuel cells, which possess high theoretical energy density and low cost.^[^ ^18^, ^19^, ^20^, ^21^ ^]^ Therefore, developing stable and efficient electrocatalysts for the OER is vital to decrease the kinetic barriers and enhance the energy utilization efficiency of related reactions and devices. From another perspective, the structural evolution of the best alkaline NiFe catalysts reported during the OER process is not fully understood,^[^ ^22^, ^23^, ^24^ ^]^ which is significant for the rational design of catalysts to obtain a mechanistic understanding of the electrocatalytic reaction in virtue of advanced in situ characterizations.

**Figure 2:**
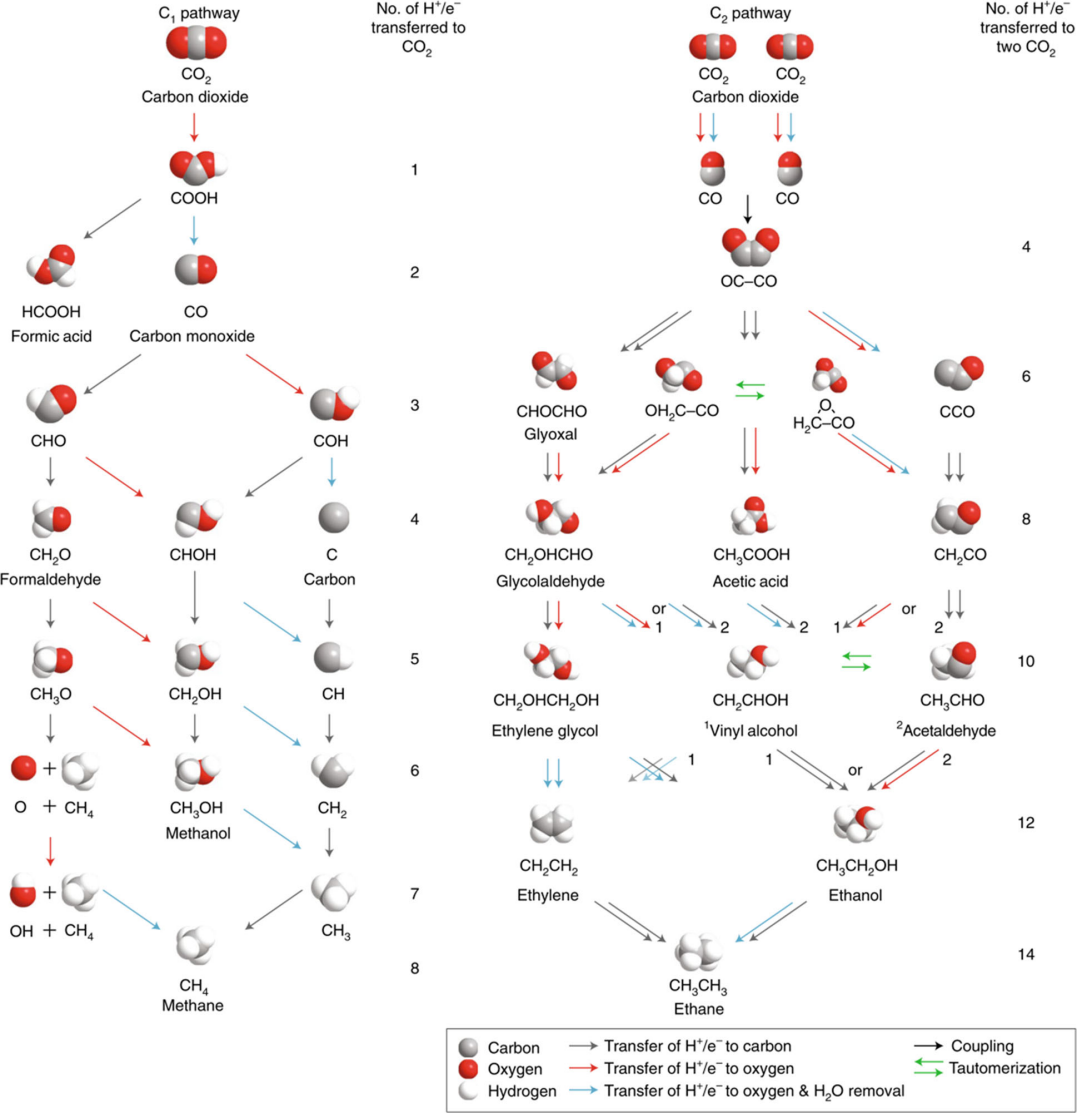
Variety of pathways displaying possible CO_2_RR routes to form single carbon products and multiple carbon products. Pathways toward a) single carbon products (via COOH and CO intermediates) and b) multiple carbon products (via CO coupling). Reproduced with permission.^[^ ^26^ ^]^ Copyright 2018, Springer Nature.

The worldwide unrestricted emissions of CO_2_ have generated serious climate change issues. The electrocatalytic CO_2_RR is regarded as an emerging technology to reduce CO_2_ emissions and simultaneously acquire high‐value‐added products.^[^ ^25^ ^]^ However, it is quite complex because of the similarity of the carbon dioxide reduction potentials of the different products. Moreover, it comprises a series of multiple electron and proton transfer steps, including numerous reaction intermediates and products.^[^ ^26^ ^]^ The CO_2_RR initially starts with the formation of CO_2_* on the catalyst active site via adsorption. Different intermediates and products are subsequently generated by electron or proton transfer.^[^ ^27^ ^]^ As shown in **Figure** **2**a, the CO_2_RR exhibits feasible pathways that the CO_2_ molecule may take during its formation of single‐carbon products via the COOH/CO pathway. The reaction mechanisms for the formation of multiple carbon products are complicated, as there are more possible reaction intermediates and pathways. Figure 2b shows the possible pathways for the formation of multiple carbon products via the OC–CO pathway. The bottleneck of CO_2_ reduction for commercial applications hitherto lies in the development of a proficient catalyst that can realize both high current density and Faraday efficiency (FE) toward multiple carbon products. Only the Cu catalyst can catalyze CO_2_ reduction to C_2_ products.^[^ ^28^ ^]^ Many researchers are dedicated to improving the catalytic performance of Cu‐based catalysts to obtain multiple carbon products.^[^ ^29^, ^30^ ^]^ Sargent and co‐workers reported a crystal facet engineering strategy to modulate Cu electrocatalysts to achieve a 90% FE for C_2+_ products at more than a partial current density of 500 mA cm^−2^.^[^ ^29^ ^]^ Due to the CO_2_ reduction complexity and product distribution diversity, researchers worldwide are working on utilizing advanced operando techniques to unveil the plausible CO_2_ reduction mechanism under working conditions.^[^ ^26^ ^]^ These operando techniques are expected to provide important experimental evidence on the configuration of key intermediates, probable reaction pathways, and product selectivity.

Operando spectroscopy entails real‐time measurement of the entire electrocatalytic process, such as infrared (IR) spectroscopy, Raman spectroscopy, and X‐ray absorption spectroscopy (XAS). Although conventional IR spectroscopy could provide clues on the chemical nature of adsorbed species on electrocatalysts, it is not suitable for electrocatalytic water splitting and CO_2_RR systems due to the serious interference of water molecules.^[^ ^26^ ^]^ In contrast, Raman spectroscopy is suitable for in situ/operando investigations of electrocatalytic water splitting and the CO_2_RR^[^ ^31^ ^]^ because it is able to effectively provide fingerprinting information of the electrocatalyst structures and reaction intermediates under working conditions.^[^ ^32^ ^]^ Further, Raman spectroscopy also possesses a high response to the vibrations derived from polarization changes and exhibits a low scattering cross‐section for water.^[^ ^33^ ^]^ However, the conventional Raman technique cannot uncover the electrochemical process occurring in nanometer‐scale catalysts due to its limited spatial resolution.^[^ ^34^ ^]^

Metallic‐based electrocatalysts and reaction intermediates with low scattering cross‐sections are not detected clearly via Raman spectroscopy. Moreover, Raman spectroscopy is only a qualitative technique.^[^ ^35^ ^]^ XAS is an alternative technique that has been utilized for in situ/operando investigation of catalysts. The advantage of XAS is its ability to provide detailed spectroscopic information on the electronic structures and coordination environments of the catalyst. In situ/operando XAS can reveal the identity of the active components, structural evolution of the catalysts, and reaction intermediates under real‐time reaction conditions.^[^ ^1^, ^36^, ^37^, ^38^ ^]^ Another advantage of XAS is that it can focus on one specific element, which is necessary to distinguish different elements in multicomponent composites. In addition, new requirements for instrumentation development are proposed to meet the in situ/operando XAS studies.

This Review is different from the previous reviews by Li and co‐workers on precise synthesis and structure characterization of electrocatalysts for water splitting,^[^ ^14^ ^]^ Deng and Yeo on using in situ Raman spectroscopy to investigate electrocatalytic water splitting and the CO_2_RR,^[^ ^31^ ^]^ and Yang and co‐workers on various in situ spectroscopy techniques for NiFe OER catalyst characterizations.^[^ ^24^ ^]^ It builds upon the recent work by Abruña et al. on the use of the operando X‐ray technique and atomic‐scale scanning transmission electron microscopy (STEM) for electrode–electrolyte interfaces.^[^ ^39^ ^]^ In this Review, we present the recent progress of in situ/operando XAS in electrocatalytic water splitting and CO_2_RR systems. In particular, we emphasize the feasibility of 1) determination of the active components, 2) probing the structural evolution of the catalyst surface, and 3) identification of reaction intermediates, highlighting the importance of innovative in situ XAS characterization. Finally, future challenges and perspectives are explored to provide guidance for investigating water splitting and CO_2_RR electrocatalysis by utilizing XAS.

A thorough understanding of the potential reaction mechanisms occurring on a catalyst's surface will help realize a reasonable design of stable and highly efficient catalysts. Previous in situ studies of single‐crystal electrodes, investigating the catalytic performance of different crystal facets, have provided vital information on small‐molecule electrocatalysis.^[^ ^40^, ^41^ ^]^ However, most catalytic materials are nanoscale and multicomponent. In addition, the surface of the catalyst undergoes reconstruction during the catalytic reaction. In the future, novel spectroscopic methodologies are expected to become more powerful as they shed light on the behavior of nanocatalysts under real working conditions. These spectroscopic methodologies have the power to dynamically monitor electrocatalysts under actual electrolytic cells.^[^ ^26^, ^42^ ^]^ It is paramount that these techniques integrate surface sensitivity and element specificity. However, each spectroscopic methodology has its own characteristics and shortcomings and cannot cover all functions.

## 2 XAS

In addition to elastic scattering and inelastic scattering, X‐ray absorption is the main consequence of the interaction between X‐rays and materials. XAS is a promising technique for exploring the electronic structures as well as local coordination structures of catalysts.^[^ ^36^ ^]^ X‐ray diffraction (XRD) including single crystals and powders is similar in this aspect, but it requires long‐range order and is unsuitable for amorphous materials. X‐ray photoelectron spectroscopy (XPS), together with other electron spectroscopies (e.g., Auger and electron energy loss spectroscopy), can provide the electronic state but must be conducted under ultrahigh‐vacuum conditions. XAS can provide geometric information on catalysts, regardless of whether they are ordered.

**Figure 3:**
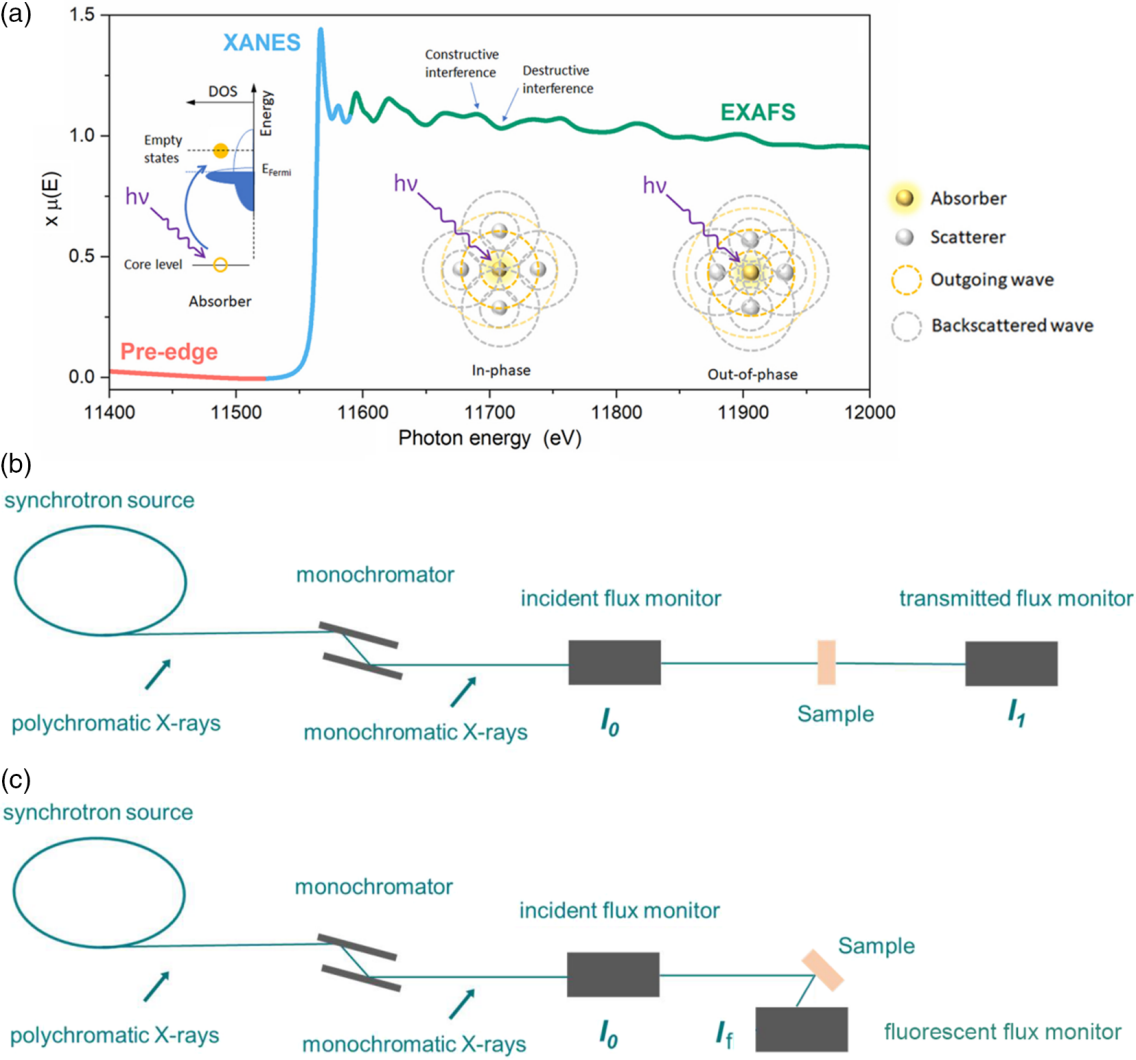
a) An XAS spectrum comprising three regions: pre‐edge (red), XANES (cyan), and EXAFS (green). The insets show the electron is excited and jumps from the core occupied state to the empty state and the interference between the outgoing photoelectron wave and backscattered photoelectron wave in the EXAFS. Reproduced with permission.^[^ ^38^ ^]^ Copyright 2021, Elsevier. Schematic diagram of b) transmission and c) fluorescence mode for measuring XAS.

XAS exhibits a functional relationship between the X‐ray absorption coefficient of a specific element and the photon energy. XAS contains X‐ray absorption near edge spectra (XANES) and extended X‐ray absorption fine structure (EXAFS). The former, extending up to 100 eV from the absorption edge, could provide information on the valence state and the electronic configuration of the specific elements in catalysts, similar to XPS. Meanwhile, the latter, extended up to 1 keV from the absorption edge, presents the information of interatomic distance and the coordination number in different shells. The physical essence of XANES and EXAFS is the photoelectron effect. However, some simplified approximations are applied in the EXAFS spectra, which allow a quantitative analysis. XANES and EXAFS provide complementary information. The local coordinated environment of each type of atom in a catalyst can be identified so that the geometric structures of the metal centers and ligands can be confirmed. Thus, XAS is an effective technique for ex situ or in situ exploration of the structural and chemical variation of the electrocatalysts during the catalytic reaction. In terms of gathering the spectra, there are different detection modes, including transmission and fluorescence. In the transmission mode, the contents of the element to be detected must be sufficiently high to achieve an acceptable signal‐to‐noise ratio. In contrast, the fluorescence mode requires low contents of elements and thick samples that cannot be transmitted via X‐rays. Both of these detection modes can obtain bulk information. **Figure** **3**b,c shows a schematic diagram for XAS measurements consisting of a synchrotron storage ring, monochromator, sample, and monitor.

### 2.1 XANES

A typical X‐ray absorption spectrum is shown in Figure 3a. An electron is excited from the core‐occupied states to the empty states after X‐ray absorption. Based on the energy difference of the emitted photoelectrons, the spectra include two different regions (i.e., XANES and EXAFS). XANES provides information on the perturbations in the valence state upon the transition of electrons from the core level, and it generates information on the hybridization of orbitals. Electron transitions are determined by the selection rule, which are dipole‐allowed and dipole‐forbidden. The valence states of the specific elements in the catalysts can be qualitatively verified by comparing the shift of the absorption‐edge position with standard samples.^[^ ^43^ ^]^ In the case of mixed valence states, a linear fitting of the first derivative of the absorption edge is used to quantitatively predict the average valence state. The shape and intensity of the metal L‐edge and O K‐edge XANES spectra in perovskites can reveal subtle information on the symmetry and spin state.^[^ ^44^ ^]^

### 2.2 EXAFS

EXAFS is the oscillation derived from the interference between the outgoing and the backscattered photoelectron waves around the absorbed atom within the photon energy range of 1000 eV above the absorption edge. Adjacent coordination atoms cause backscattering of the outgoing photoelectrons. Thus, EXAFS spectra can show the local structural information of the catalysts, including the bond distance and coordination number of neighboring atoms surrounding the absorbed atoms. In essence, the electronic and geometric structures of the catalyst can be revealed via ex situ XAS.^[^ ^45^ ^]^ There are two types of XAS depending on the photon energy of the incident X‐ray: hard X‐rays (>2 keV) and soft X‐rays (<2 keV).^[^ ^46^, ^47^ ^]^ Soft XAS has been widely utilized for probing the structures of light elements (B, C, N, O, S, and P) in catalysts. Thus, metal and nitrogen codoped carbon catalysts could be investigated utilizing soft XAS.

The ex situ metal K‐edge XAS is an effective tool to study the electronic structure of electrocatalysts. Sometimes it cannot distinguish the differences in XAS between active and inactive centers.^[^ ^48^ ^]^ To comprehend the structure–activity relationship, it may be required that metal L‐edge and O K‐edge XAS is utilized. The metal L‐edge is the excited transition from the 2*s*/2*p* core level to the 3*d* level, which can detect the unoccupied 3*d* state.^[^ ^49^ ^]^ The performances of transition metal‐based catalysts are greatly affected by the 3*d* orbital occupied/unoccupied states. In addition, O K‐edge XAS can uncover hybridization of transition metal 3*d* and oxygen 2*p* states in oxides for the OER.^[^ ^50^ ^]^ Specifically, the pre‐edge feature of the O K‐edge XAS can be used to predict the trend in hybridization, which significantly affects the OER performance of transition metal oxide‐based catalysts. The hybridization increases with the oxidation of the transition metal, which reduces the energy difference between the transition metal 3*d* and oxygen 2*p* to enhance the covalency.^[^ ^51^, ^52^, ^53^ ^]^ In addition, the orbital–lattice interaction derived from strained engineering and magnetic catalysts can be investigated via X‐ray linear dichroism (XLD) measurement.^[^ ^54^ ^]^ Ex situ XAS can characterize the structural information of a catalyst before and after the electrocatalytic reaction, from which to indirectly predict possible change in the electrocatalytic reaction.

## 3 In Situ XAS

Sophisticated characterization techniques are imperative to further understand the underlying mechanism of electrocatalysis. Although ex situ XAS can be utilized to investigate the electronic and geometric structures of catalysts, probing mechanisms of catalytic reactions are not feasible, specifically for monitoring the dynamic change of metal catalytic centers in real time. In the past decade, in situ XAS has been used to systematically investigate the mechanism of electrocatalytic water splitting and the CO_2_RR. In the meantime, in situ XAS instruments for electrochemical testing are developing rapidly,^[^ ^55^ ^]^ which primarily consist of an X‐ray spectrometer and a unique electrochemical cell. Using the high energy and penetration characteristics of synchrotron radiation X‐rays, the in situ technique can provide insight into the electrochemical reaction at the atomic level.^[^ ^56^, ^57^, ^58^ ^]^ Furthermore, state‐of‐the‐art nitrogen‐ and carbon‐immobilized transition metal single‐atom catalysts have been extensively investigated by the in situ XAS technique based on soft X‐ray.^[^ ^58^, ^59^ ^]^

### 3.1 In Situ XAS Instruments for Electrocatalysis

**Figure 4:**
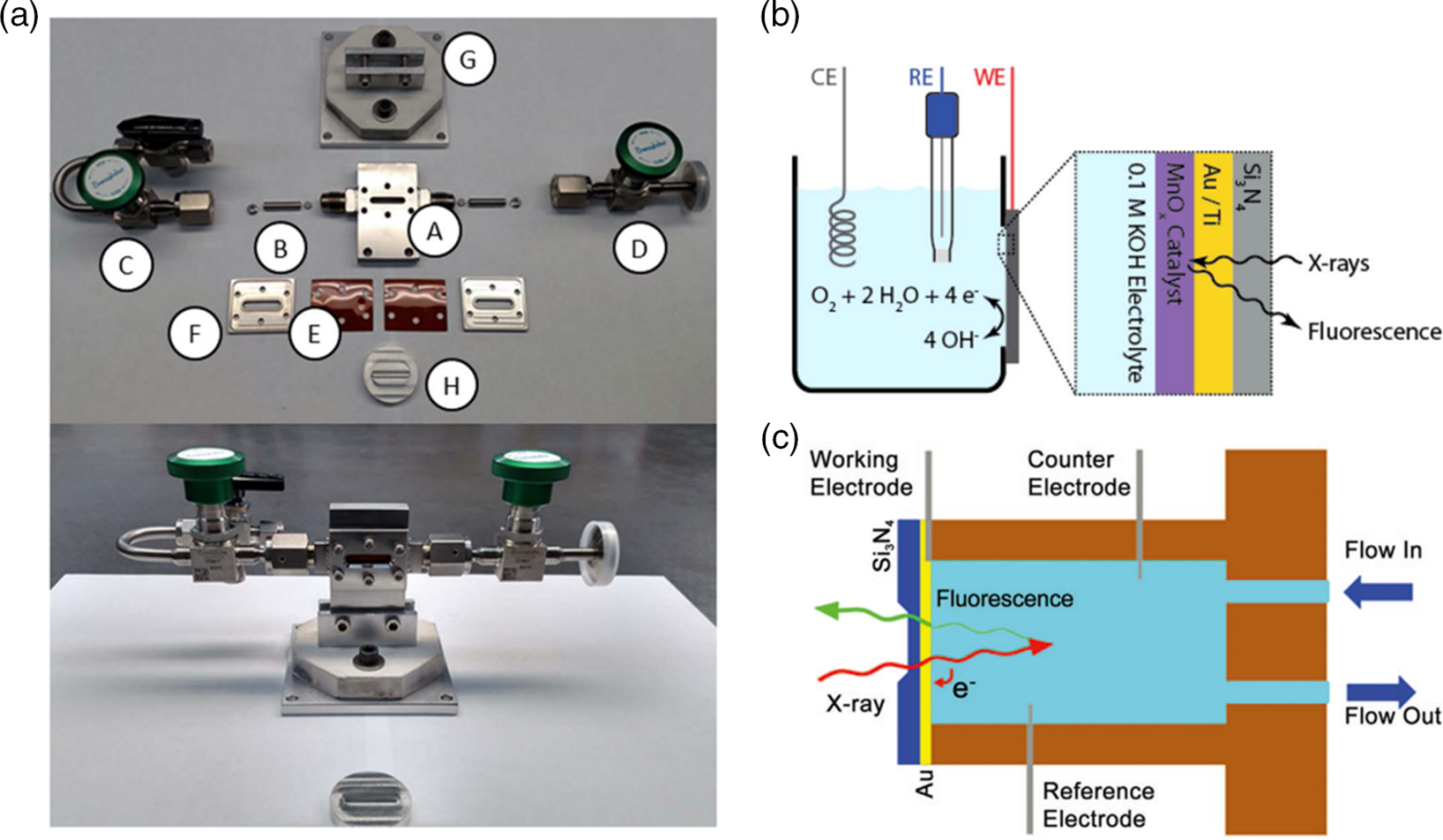
a) Unassembled XAS cell (top) and assembled XAS cell with sample press (bottom). Reproduced with permission.^[^ ^60^ ^]^ Copyright 2016, AIP Publishing. b) In situ XAS setup and the electrolyte in contact with the front side of a Si_3_N_4_ window with electrodeposited MnO_*x*_ on a layer of Au/Ti and with the back side of the window facing the X‐rays. Reproduced with permission.^[^ ^61^ ^]^ Copyright 2013, American Chemical Society. c) Electrochemical cell for soft XAS in fluorescence mode. Reproduced with permission.^[^ ^62^ ^]^ Copyright 2014, American Association for the Advancement of Science.

Recent developments in in situ XAS instruments for electrocatalysis have focused on the rational design of electrochemical cells and their related components. The cell consists of four sections: the body, inlet and outlet plumbing, windows, and stage/cell holder (**Figure** **4**a). The electrochemical cell in water splitting can be conducted in two patterns according to the detection modes: transmission and fluorescence, as shown in Figure 4b,c. Among them, the transmitted X‐rays are collected rapidly during the testing process. However, the thickness of the sample must be sufficiently thin to allow transmission. In addition, the detection of transmitted X‐rays requires a large number of catalysts. In contrast, the fluorescence X‐rays can be detected regardless of the thickness and amount of catalyst, generating a highly improved element sensitivity.^[^ ^60^ ^]^ Figure 4b shows an experimental setup of an in situ X‐ray electrochemical cell via transmission mode.^[^ ^61^ ^]^ The setup consists of an X‐ray transparent window (made of Si_3_N_4_) with the catalyst deposited on an Au/Ti conductive layer adhered onto the window as the working electrode. The two sides of the window face the electrolyte body and ray source, respectively. Although X‐rays can pass through the window and electrolyte, the thickness of the window and that of the conductive layer are crucial factors in influencing the attenuation degree of the X‐rays. Figure 4c shows the experimental setup of the in situ XAS electrochemical cell via fluorescence mode.^[^ ^62^ ^]^ In view of the drawbacks of the transmission mode, the fluorescence pattern of in situ XAS is preferable for practical electrocatalytic reactions. However, the in situ XAS flow cell is utilized in the CO_2_RR. It includes a CO_2_ gas chamber and two liquid chambers containing catholyte flow and anolyte flow chambers on either side of an anion exchange membrane. The gas and catholyte chambers are separated by gas diffusion electrodes (GDEs), which provide plentiful of three‐phase boundaries across the electrode–electrolyte interface. In view of the attenuation of hydrophobicity of GDEs in the CO_2_RR under alkaline media, there is another catholyte‐free MEA (membrane electrode assembly) system incorporating two titanium backplates with a serpentine flow field and the MEA. The MEA comprises a GDE cathode and anode on either side of the anion exchange membrane. The gap between the electrodes was minimized to reduce Ohmic losses compared to the flow cell.^[^ ^63^, ^64^ ^]^ By utilizing the in situ XAS apparatus, the dynamic change of the catalysts’ surface can be elaborately tracked throughout the electrocatalysis process.

## 4 In Situ XAS for Electrocatalytic Reactions

### 4.1 In Situ XAS for Water Splitting

**Figure 5:**
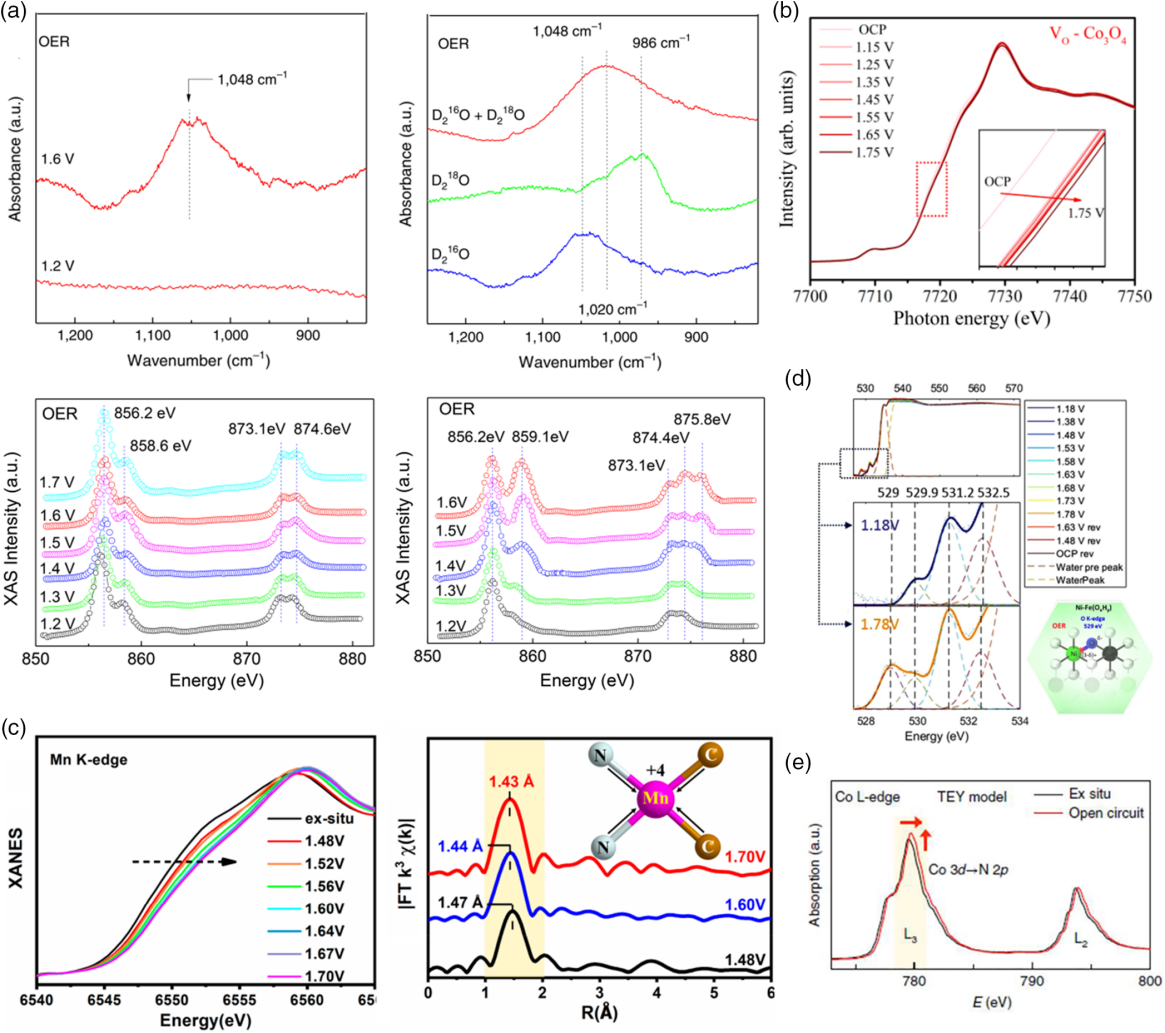
a) Operando SR‐FTIR spectroscopy measurements and isotope‐labeling operando SR‐FTIR spectroscopy measurement for the NiFe MOF, XAS measurements of Ni *L*_3,2_‐edge for pristine and lattice‐strained NiFe MOF during the OER, respectively. Reproduced with permission.^[^ ^65^ ^]^ Copyright 2019, Springer Nature. b) Operando XAFS for Co K‐edge of Vo–Co_3_O_4_; insets show a detailed view of the dotted boxes. Reproduced with permission.^[^ ^66^ ^]^ Copyright 2020, American Chemical Society. c) Operando XAFS characterization of Mn single‐atom catalyst: Mn K‐edge XANES spectra and EXAFS spectra of Mn single‐atom catalyst during the OER. Reproduced with permission.^[^ ^67^ ^]^ Copyright 2020, American Chemical Society. d) In situ XAS O K‐edge spectra of Ni–Fe catalyst. Schematic illustration showing the correlation between the changes at the O K‐edge at 529 eV and the Ni *L*‐edge at 854.1 eV. Reproduced under the terms of the CC‐BY 4.0 license.^[^ ^57^ ^]^ Copyright 2019, The Authors, published by Springer Nature. e) Co *L*‐edge XANES spectra of ex situ catalyst and under the open‐circuit condition. Reproduced with permission.^[^ ^58^ ^]^ Copyright 2019, Springer Nature.

In situ XAS often recognizes the active sites by analyzing the near‐edge position change, which indicates a change in the valence state and deviation in bond length between the metal center and coordinated atom during electrocatalysis. Typically, the atoms of interest in the electrocatalysts act as catalytic centers, while the other coordinated atoms play an assistant role in altering the properties of the concerned atoms or providing suitable surroundings for adsorbates. Liu and co‐workers synthesized a lattice‐strained NiFe metal organic framework (MOF) for bifunctional oxygen electrocatalysis via a photoinduced lattice strain strategy. The operando synchrotron radiation Fourier transform infrared (SR‐FTIR) combined with soft XAS techniques was exploited to probe the active components and reaction intermediates.^[^ ^65^ ^]^ As shown in **Figure** **5**a, the isotope‐labeling operando SR‐FTIR results confirmed that the vibration peak at 1048 cm^−1^ was attributed to the surface intermediate superoxide OOH* under an applied potential of 1.6 V versus reversible hydrogen electrode (RHE). The operando soft XAS exhibited a peak at 875.8 eV, which was ascribed to the Ni^4+^ species with the applied potential increasing up to 1.6 V versus RHE. The Ni^4+^ species disappeared when the applied potential was switched back to the initial potential of 1.2 V versus RHE. Compared with the lattice‐strained NiFe MOF, the pristine NiFe MOF did not show any characteristic peaks at 875.8 eV, despite the increase in applied potential up to 1.7 V versus RHE. Wang and co‐workers constructed a rich oxygen vacancy Co_3_O_4_ via Ar plasma as a model catalyst to investigate the dynamic evolution of oxygen vacancy sites during the OER process using operando XAS combined with other characterizations. To maintain electric neutrality, the oxygen vacancy leads to the formation of low‐valence‐state cobalt in Co_3_O_4_. Operando electrochemical impedance spectroscopy (EIS) and cyclic voltammetry (CV) revealed that the oxygen vacancies can benefit the preoxidation of Co^2+^ at a lower applied potential. Operando XAS in combination with quasi‐operando XPS confirmed that the oxygen vacancies were refilled with hydroxyl for the vacancy Co_3_O_4_ and boosted the reconstruction/deprotonation of the intermediate Co—OOH (Figure 5b).^[^ ^66^ ^]^ Li and co‐workers developed a carbon/nitrogen‐immobilized Mn single catalyst for oxygen electrocatalysis through interface engineering. Through operando XAS, the authors revealed that the bond‐distance‐stretched Mn^2+^—N_2_C_2_ atomic interface sites acted as catalytic sites in the ORR, while the bond‐distance‐contracted Mn^4+^—N_2_C_2_ moieties served as the active centers for the OER.^[^ ^67^ ^]^ Mn K‐edge XANES spectra showed that the XANES edge was shifted toward higher energies after a series of positive potentials was applied to the working electrode, implying an increase in the Mn valence state. The EXAFS spectra demonstrated that the peak position of Mn—N/C displayed a clearly low‐*R* shift from 1.47 to 1.43 Å for the OER (Figure 5c). Theoretical calculations confirmed that the geometric structure of the two nitrogen and two carbon atom immobilized one Mn atom was the most stable compared with the other three structures.

The frontier orbitals (*nd* orbitals for transition metal cations and 2*p* orbitals for C/N/O anions) can be investigated via in situ soft XAS. The entire experiment must be conducted in an ultrahigh‐vacuum environment to avoid unnecessary loss of the incident soft X‐ray with low energy. In situ O K‐edge XAS spectra are divided into three regions: 1) the energy range of 525–534 eV, which corresponds to the electronic transition from O 1*s* to O 2*p* M^−1^ 3*d* ^[^ ^51^ ^]^; 2) of 534–540 eV, which exhibits the near‐edge characteristics of water molecules; and 3) above 540 eV, which shows the EXAFS of the O K‐edge. Lange et al. utilized the in situ O K‐edge XAS to unveil the local bonding states and symmetry feature of the O anion in electrodeposited NiFeO_*x*_H_*y*_ OER electrocatalysts.^[^ ^57^ ^]^ As shown in Figure 5d, four peaks at 529, 529.9, 531.1, and 532.5 eV were identified as the transitions from O 1*s* to O 2*p* hybridized with Ni 3*d t*_2*g*_, Fe 3*d* *t*_2*g*_, Oπ^*^ of O_2_ gas, and Fe 3*d* *e*_*g*_, respectively.^[^ ^68^ ^]^ The peak at 529 eV was the O 1*s*–O 2*p*/Ni 3*d* *t*_2*g*_ transition that appeared at the OER potential of 1.78 V due to an increase in the valence state of Ni and receded reversibly at the reductive potential of 1.18 V. The variation of this peak was related to a change in hybridization between O 2*p* and Ni 3*d*, resulting from the injection or extraction of electrons from O to Ni sites (${\text{Ni}}^{3+}-O^{2-}↔{\text{Ni}}^{\left(3-\delta \right)+}-O^{\left(2-\delta \right)-}$). In contrast, the peaks at 529.9 eV (O 1*s*–O 2*p*/Fe 3*d* *t*_2*g*_) and 532.4 eV (O 1*s*–O 2*p*/Fe 3*d* *e*_2*g*_) exhibited slight changes with the applied voltage, demonstrating that the Fe sites underwent little variation compared to Ni sites. The in situ O K‐edge uncovered the orbital hybridization of O 2*p* with transition metal ions and charge transfer between them, which revealed the evolution of the frontier orbitals during the electrocatalysis process.

The valence state change and charge transfer due to adsorbed intermediates for the metal nitrogen–carbon electrocatalyst can also be confirmed by in situ soft XAS. Wei and co‐workers constructed an atomically dispersed cobalt catalyst in the framework of phosphorized carbon nitride (PCN) via nitrogen immobilization for alkaline HER. The dynamic structures of the cobalt single‐atom catalyst active sites were identified using operando XAFS.^[^ ^58^ ^]^ As shown in Figure 5e, the ex situ catalyst was the Co_1_/PCN coated on conductive silicon and immersed in KOH aqueous solution (HO–Co_1_/PCN) under open circuit voltage. The charge density difference between Co_1_/PCN and HO–Co_1_/PCN revealed that the Co atom primarily contributed its electrons to its surrounding N atoms and the adsorbed OH^−^ through orbital hybridization, which could be reflected by the Co K‐edge and L‐edge XANES spectra. The valence states of the Co atoms further increased with increasing applied bias voltage. Furthermore, the valence states of Co were evaluated by normalizing the Bader charges to standard samples. The nominal valence states of ex situ Co_1_/PCN and open‐circuit HO–Co_1_/PCN samples were 2.05 and 2.16, respectively, which was consistent with the previous XANES results. These results proved the higher valence state of Co atoms under working conditions and validated the strong charge transfer occurring on HO–Co_1_–N_2_, which can accelerate the sluggish kinetics of the Volmer step in alkaline HER, thereby improving the catalytic activity.

### 4.2 In Situ XAS for CO_2_RR

**Figure 6:**
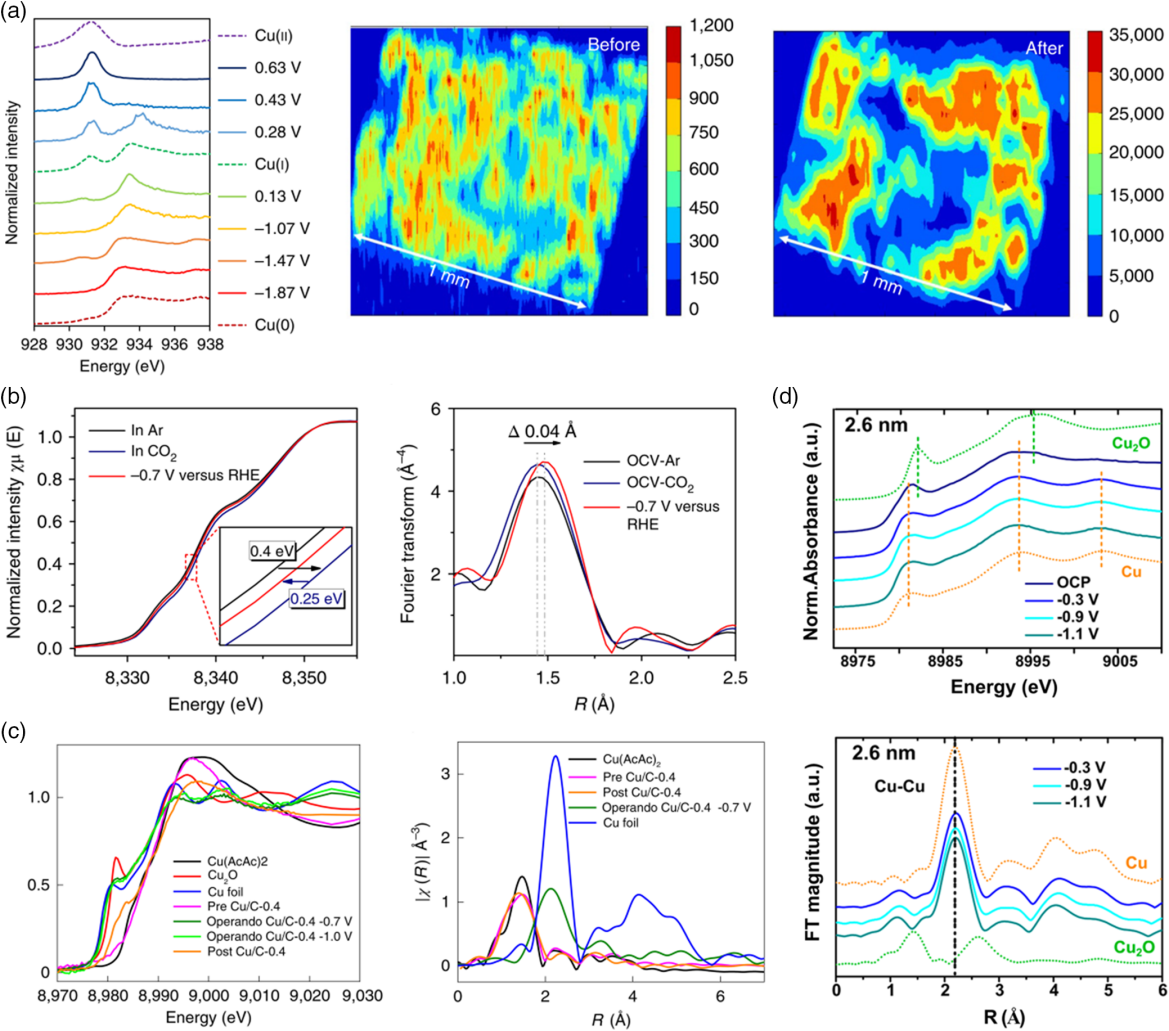
a) In situ Cu *L*_3_‐edge soft XAS spectra of Cu catalyst (solid lines) and ex situ soft XAS spectra of reference standards (dotted lines) and soft XAS 2D mapping of the Cu intensity before and after the CO_2_RR. Reproduced with permission.^[^ ^56^ ^]^ Copyright 2018, Springer Nature. b) Normalized operando Ni K‐edge XANES spectra and Fourier transform magnitudes of EXAFS spectra of A–Ni–NG. Reproduced with permission.^[^ ^71^ ^]^ Copyright 2018, Springer Nature. c) In situ Cu K‐edge XANES spectra of Cu catalysts, Fourier transform of *k* ^2^‐weighted *χ* function in *R* space of the Cu catalysts. Reproduced with permission.^[^ ^72^ ^]^ Copyright 2020, Springer Nature. d) In situ GIXAS of Cu(pc) catalyst and XANES and EXAFS spectra of the Cu(pc) catalyst at a probe depth of 2.6 nm under different applied potentials. Reproduced with permission.^[^ ^73^ ^]^ Copyright 2021, American Chemical Society.

Electrocatalytic water splitting solely generates the desired hydrogen or oxygen through a two‐ or four‐electron process, respectively. However, the CO_2_RR requires a much higher applied bias voltage and involves a multielectron transfer process. Sargent and co‐workers designed an electro‐redeposited (ERD) copper catalyst to enhance the ethylene selectivity of CO_2_ electrocatalytic reduction via the dissolution and redeposition of copper from a sol–gel process.^[^ ^56^ ^]^ The valence state variation of the copper catalyst in the CO_2_ reduction process was tracked in real time via in situ soft XAS. The valence state of copper was probed by the in situ Cu *L*_3_‐edge under different applied bias voltages, as shown in **Figure** **6**a. A distinct peak at 931 eV corresponding to Cu^2+^ was observed at applied potentials more positive than 0.28 V versus RHE. The transformation of Cu from Cu^2+^ to Cu^+^ occurred at 0.28 V versus RHE, which was reflected by the decrease in the peak intensity at 931 eV. While the applied potential was 0.13 V versus RHE, the predominant valence state of copper was +1. As the negative potential was −1.87 V versus RHE, the spectrum perfectly matched with that of standard sample copper, demonstrating a complete transformation from Cu^+^ to Cu^0^. The as‐obtained in situ spectra fitted through a linear combination confirmed the existence of Cu^+^ at the negative potential of −1.47 V versus RHE under CO_2_RR conditions. Soft XAS 2D contour maps of the Cu intensity demonstrated that the Cu signal was distributed homogeneously across the sample before the reaction and highly localized in certain regions after reduction, implying some degree of aggregation. Theoretical calculations demonstrated that the formation energy of the methane intermediate (COH*) was much higher than that of the ethylene intermediates (OCCOH*) on the surface of the Cu^+^ species. The in situ XAS combined with theoretical calculations revealed that copper oxychloride sol–gel stabilized Cu^+^ species efficiently improved the selectivity of ethylene for electrocatalytic CO_2_ reduction.

Nitrogen‐ and carbon‐immobilized transition metal single‐atom catalysts have shown outstanding activities toward various small‐molecule electrocatalyts. However, the atomically dispersed metal atoms are easy to agglomerate due to their high surface energy and migration energy, which greatly limits their practical application. A key advantage of XAS is its ability to detect the structural information of catalysts at the atomic level.^[^ ^69^, ^70^ ^]^ Huang et al. designed a single‐atom catalyst involving monovalent Ni(1) anchored on nitrogen‐doped graphene for efficient CO_2_RR and unveiled the specific adsorption and activation process of CO_2_.^[^ ^71^ ^]^ The electronic states of Ni in single‐atom catalysts were first confirmed by ex situ Ni K‐edge XANES spectra. As shown in Figure 6b, for the operando XAS results, a blueshift of the Ni K‐edge (0.4 eV) was observed in the CO_2_‐saturated KHCO_3_ solution compared to that in the Ar‐saturated solution under open‐circuit voltage, indicating an increase in the Ni valence state. This can be ascribed to the delocalization of the unpaired electrons in the 3*d* orbital of Ni^+^ and the spontaneous charge transfer from the central Ni^+^ to the carbon 2*p* orbital of CO_2_ to form a ${\text{CO}}_{2}^{\delta -}$ intermediate. After switching to the bias voltage of −0.7 V versus RHE, the Ni K‐edge re‐shifted ≈0.25 eV back to the low energy and implied the partial recovery of the low‐oxidation state. This relatively small change in the valence state of Ni ions implies that the adsorbed ${\text{CO}}_{2}^{\delta -}$ intermediates underwent one CO_2_RR cycle, thus restoring the low‐valence‐state nickel ion during the CO_2_RR. In situ EXAFS spectra revealed that the intensity of the main peak increased to a certain extent, contributed by the Ni—C bond due to the chemical adsorption of CO_2_ on the nitrogen‐coordinated Ni ion. The main peak moving toward the direction of increasing bond length (0.04 Å) at a biased voltage of −0.7 V versus RHE during the CO_2_RR demonstrated attenuation of the Ni—N bond. This implied that the electrons in the Ni 3*d* orbital coordinated with four nitrogen atoms (Ni—N) were redistributed and the central Ni ions were distorted out of the graphene plane. Xu and co‐workers developed a supported Cu catalyst via an amalgamation strategy to realize a multiple‐carbon product FE of 91% at −0.7 V versus RHE and a low onset potential of −0.4 V versus RHE for conversion of carbon dioxide to ethanol. Operando hard XAS uncovered the existence of a reversible transformation between atomically dispersed Cu atoms and Cu_*n*_ clusters (*n* = 3 and 4) under electrochemical conditions.^[^ ^72^ ^]^ The electronic and geometric structural changes of Cu in Cu/C–0.4 were investigated using operando hard XAS during CO_2_RR. The Cu K‐edge XANES spectra predominantly resembled that of Cu^0^ species containing a small amount of Cu^+^ when the cell voltage was applied at −0.7 V versus RHE (Figure 6c). The EXAFS results showed that the Cu first‐shell coordination was transformed from Cu—O to Cu—Cu under the electrocatalytic CO_2_ reduction process, in which the coordination number increased from 2(±0.9) to 3(±1.2), implying the formation of an ultrasmall Cu moiety (Cu_*n*_, *n* = 3 or 4). The Cu moiety anchored by the hydroxyl from the KHCO_3_ electrolyte sequentially activated the C—O bond, hydrogenated, coupled the C—C bond, and finally realized the conversion of CO_2_ to ethanol through successive electron and proton transfer processes. When the cell voltage was removed, the first coordination shell and Cu valence state were immediately transformed from Cu—Cu to Cu—O ligation with Cu^2+^, suggesting the reoxidation of the Cu moiety to the high‐valence‐state Cu single atom in a CO_2_‐saturated electrolyte. Mehta and co‐workers unveiled the structural change of a polycrystalline Cu electrocatalyst surface under CO_2_ reduction conditions through GIXAS combined with XRD (GIXRD).^[^ ^73^ ^]^ The in situ GIXAS revealed that a preferential surface reconstruction of the polycrystalline Cu toward Cu (100) facets at the CO_2_ reduction potential and the degree of reconstruction increased with increasing applied negative potentials (Figure 6d). The in situ GIXRD demonstrated that the metallic Cu surface could be reversibly reoxidized into Cu_2_O when removing the CO_2_ reduction potential.

## 5 Conclusion

We have given a critical review of the state‐of‐the‐art XAS characterizations, specifically the in situ XAS, for a thorough understanding of electrocatalytic small‐molecule conversion, such as water splitting and CO_2_ reduction. In particular, the electrocatalytic reactions involving small molecules (HER, OER, and CO_2_RR) are crucial for energy conversion utilization. However, the catalytic origin of these reactions has not been elucidated due to the difficulty in studying the structural changes during electrocatalysis. In situ XAS provides powerful evidence for probing the in‐depth mechanism of electrocatalytic water splitting and the CO_2_RR, including the recognition of the active component, tracking the dynamic structural evolution of the electrocatalysts, and observation of steady reaction intermediates in nitrogen/carbon‐immobilized transition metal single‐atom catalysts, transition metal oxides, and metal polycrystals.

A single technique cannot provide full‐scale details of processes such as water splitting and CO_2_RR complex catalytic systems, and XAS is without exception. The conventional XAS technique hitherto requires minute‐level collection of a set of XAS spectra. This relative low‐speed acquisition of data cannot allow for real‐time analysis of the kinetic process. Thus, it is necessary to develop time‐resolved in situ XAS to monitor the structural changes in the catalyst. XAS is generally insensitive to lighter elements as adsorbed species can only be detected indirectly by monitoring subtle changes in the coordination environment of the catalysts.^[^ ^67^ ^]^ Moreover, there remain several technical issues, such as the exploitation of time‐resolved soft X‐ray techniques for monitoring the oxygen intermediates in the OER. Future progress relies heavily on innovations in operando XAS techniques. Resonant‐inelastic X‐ray scattering (RIXS) can be regarded as one of the prospective techniques for distinguishing different oxygen intermediates, such as OH*, O*, and OOH* originating from oxygen electrocatalysis. In principle, RIXS spectra can be obtained by the continuous excitation of electrons and emission of photons. In addition, XAS is unsuitable for distinguishing metal–X (X=C, N, O) bonds in M–N–C catalysts due to the similar X‐ray scattering capability of the light elements and cannot reveal the local geometric structure of inhomogeneous metal alloys. In this regard, theoretical fitting of XANES spectra can exclude certain impossible configurations.^[^ ^74^ ^]^ On this basis, X‐ray emission spectroscopy (XES) can further confirm the reasonable geometric configuration of the metal center and coordinated atoms.^[^ ^75^ ^]^ Moreover, utilizing the total scattering atomic pair distribution function (PDF) method could also confirm the aforementioned subtle bond length differences and structural inhomogeneity of alloy catalysts.^[^ ^76^, ^77^ ^]^ Furthermore, XAS signals can be used for 3D chemical tomography imaging of catalysts via computer postprocessing during the electrocatalytic reactions. Operando computed tomography (CT) coupled with XANES unveiled the degradation phenomenon‐of a polymer electrolyte fuel cell. The primary reason is the migration of the Pt catalyst into the Nafion membrane, resulting in a short circuit.^[^ ^78^ ^]^ In summary, we believe that the further development of XAS coupled with other operando techniques (e.g., Raman spectroscopy, nuclear magnetic resonance. and Mössbauer), in combination with theoretical calculations, will lead the way toward deeper comprehension of the fundamental processes of water splitting and the CO_2_RR.

## Conflict of Interest

The authors declare no conflict of interest.

## Data Availability Statement

Data sharing not applicable to this article as no datasets were generated or analyzed.
